# Exosomes from adipose‐derived stem cells and application to skin wound healing

**DOI:** 10.1111/cpr.12993

**Published:** 2021-01-17

**Authors:** Yang An, Shuyan Lin, Xiaojie Tan, Shiou Zhu, Fangfei Nie, Yonghuan Zhen, Luosha Gu, Chunlei Zhang, Baicheng Wang, Wei Wei, Dong Li, Junhao Wu

**Affiliations:** ^1^ Department of Plastic Surgery Peking University Third Hospital Beijing China; ^2^ Hearing Ctr Department of Otolaryngology, Head and Neck Surgery West China Hospital Sichuan University Chengdu China; ^3^ State Key Laboratory of Biotherapy and Cancer Center West China Hospital Sichuan University Chengdu China; ^4^ Institute of Systems Biomedicine Peking University Beijing China; ^5^ Clinical Stem Cell Research Center Peking University Third Hospital Beijing China

**Keywords:** adipose‐derived stem cells, angiogenesis, exosomes, inflammation, skin wound healing

## Abstract

Skin wound healing is an intractable problem that represents an urgent clinical need. To solve this problem, a large number of studies have focused on the use of exosomes (EXOs) derived from adipose‐derived stem cells (ADSCs). This review describes the mechanisms whereby ADSCs‐EXOs regulate wound healing and their clinical application. In the wound, ADSCs‐EXOs modulate immune responses and inflammation. They also promote angiogenesis, accelerate proliferation and re‐epithelization of skin cells, and regulate collagen remodelling which inhibits scar hyperplasia. Compared with ADSCs therapeutics, ADSCs‐EXOs have highly stability and are easily stored. Additionally, they are not rejected by the immune system and have a homing effect and their dosage can be easily controlled. ADSCs‐EXOs can improve fat grafting and promote wound healing in patients with diabetes mellitus. They can also act as a carrier and combined scaffold for treatment, leading to scarless cutaneous repair. Overall, ADSCs‐EXOs have the potential to be used in the clinic to promote wound healing.

## Graphical abstract

This review describes the mechanisms whereby exosomes (EXOs) derived from adipose‐derived stem cells (ADSCs) regulate wound healing and their clinical application. In the wound, ADSCs‐EXOs modulate immune responses and inflammation, promote angiogenesis, accelerate proliferation and re‐epithelization of skin cells and regulate collagen remodelling, which inhibits scar hyperplasia. ADSCs‐EXOs have the potential to be used in the clinic, as they can improve fat grafting, promote wound healing of diabetic patients and act as a carrier and combined scaffold for treatment, leading to scarless cutaneous repair.

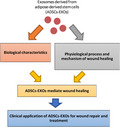

## 1 INTRODUCTION

Wound healing is a complex biological process that takes place in skin tissue after injury by trauma, burn or diabetic ulcers.^1^ Chronic skin wounds are difficult to heal, for example, in diabetic or long‐term bedridden patients.^2^ Therefore, wound healing is one of the most intractable problems for clinicians and a heavy burden for patients, both physically and financially. Conventional wound care methods, with the risk of atrophic scars and pigmentary abnormalities, include skin grafting, skin flap transplantation, laser therapy and biological stents.^3^, ^4^ Also, biological scaffolds are costly and slow, and they are not suitable to treat large scale trauma.^5^, ^6^

Other treatments include local application of specific growth factors^7^ and gene therapy.^8^ However, local growth factors are easily degraded in body fluids, whereas dosage cannot be easily controlled at the wound site.^9^ Hence, there is a crucial and urgent need for alternative efficient and safe methods to promote wound healing.

Recently, stem cell therapy has flourished because of its pluripotency, self‐renewal and the ability to promote secretion of regenerative cytokines.^10^ Pluripotent stem cells are considered safe and overcome moral concerns associated with embryonic stem cells. However, stem cell therapy may present both problems of storage and transportation and risks of induced tumorigenesis and deformity.^11^ Stem cells have been proposed to promote wound healing in a paracrine way by (1) regulating macrophages,^12^ T cells, B cells and others^13^, ^14^, ^15^ to reduce inflammation, (2) secreting VEGF to promote angiogenesis,^16^ (3) promoting proliferation and differentiation of fibroblasts and keratinocyte‐forming cells, (4) producing anti‐fibrosis cytokines and (5) transforming into microvascular endothelial cells and keratinocytes.^13^, ^17^

Exosomes are one of the components of paracrine and the main contributor to stem cells efficacy.^18^ They are small, single membranous, secretory organelles rich in proteins, lipids, nucleic acids and carbohydrate conjugates. They are also thought to have a wide variety of activities, such as remodelling the extracellular matrix and delivering signals and molecules to other cells. Their usage avoids many of the shortcomings of stem cells, since they are stable and easily stored. In addition, they are not rejected by the immune system, have a homing effect, and dosage can be easily controlled.^19^, ^20^

Exosomes derived from adipose‐derived stem cells (ADSCs‐EXOs) have become a hot topic in the field of skin wound repairing and treatment. Adipose‐derived stem cells (ADSCs) are derived from adipose tissue, where they are nearly 500 times more abundant than in an equivalent amount of bone.^21^, ^22^ The abundance and the simple methods of sampling of ADSCs make it safer against trauma and other adverse reactions.

## 2 BIOLOGICAL CHARACTERISTICS OF EXOS

### 2.1 Biogenesis and release

**FIGURE 1:**
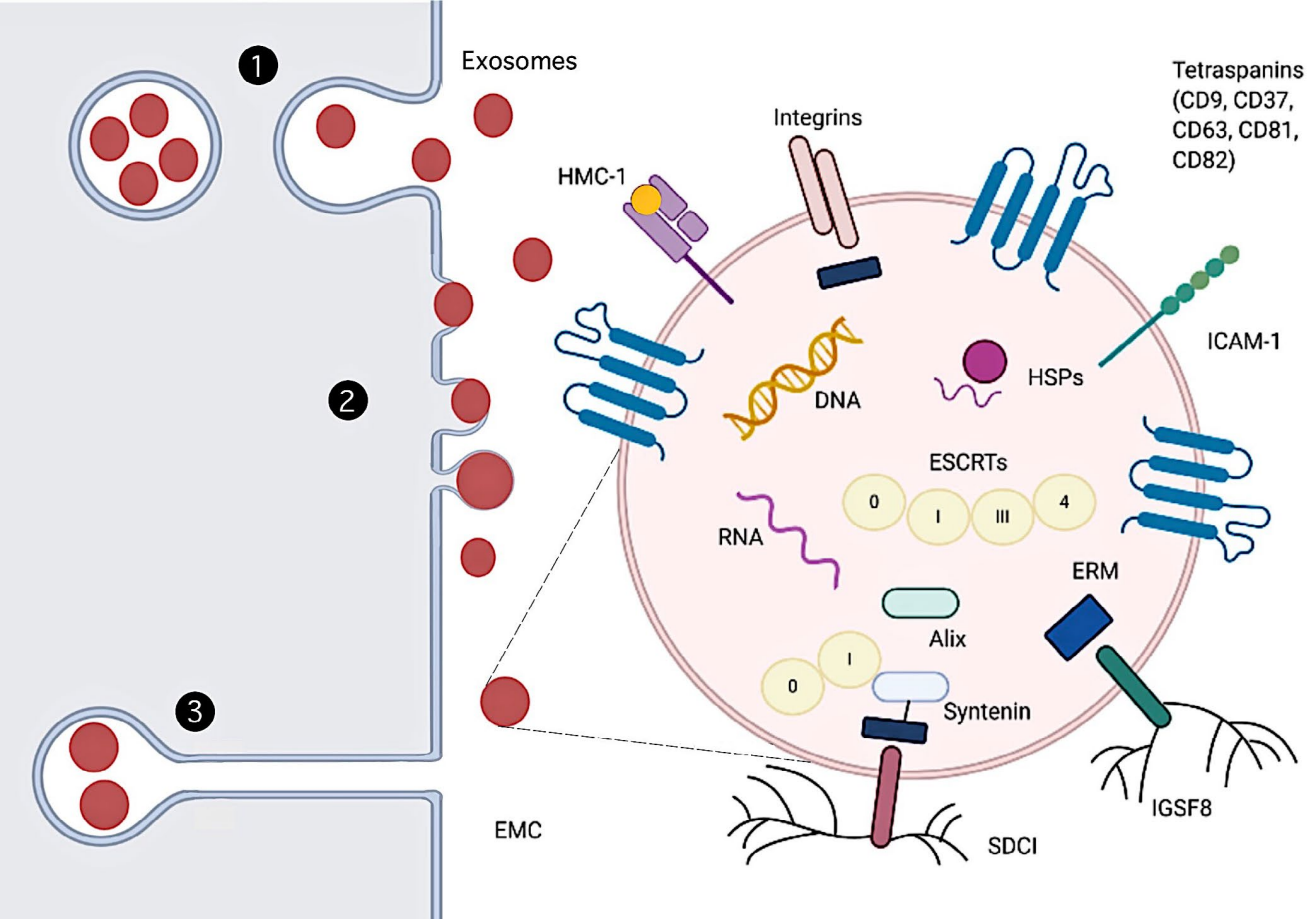
EXOs bud from endosome and plasma membranes. Exosome biogenesis may use three mechanisms: (1) vesicle budding into discrete endosomes that mature into multivesicular bodies, releasing EXOs upon plasma membrane fusion; (2) direct budding from plasma membrane and (3) delayed release by budding at intracellular plasma membrane–connected compartments (IPMCs) followed by deconstriction of IPMC neck(s). We note that this is not a comprehensive list and it is just to illustrate some of the mechanisms. Abbreviations: ECM, extracellular matrix; ERM, ezrin‐radixin‐moesin; ESCRTs, endosomal sorting complexes required for transport; MHC, major histocompatibility complex; IGSF8, immunoglobulin superfamily member 8; ICAM‐1, intercellular adhesion molecule‐1; SDC1, syndecans 1; HSPs, heat shock proteins

Exos, as a subtype of extracellular vesicles (EVs), are derived from endosome and plasma membranes through endocytosis, fusion and budding processes.^23^ There modes of EXOs and other EVs biogenesis and release are as follows (Figure 1). In the first, the primary endosomes produced by phagosomes and plasma membrane are further acidified to form secondary endosomes. These subsequently bud inward to form multivesicular bodies (MVBs). Some MVBs entering lysosomes are degraded, whereas the rest release EVs when they fuse to the plasma membrane. This last step has been shown using genetically encoded, pH‐sensitive CD63–pHluorin fusion proteins.^24^ In the second, EVs directly bud from the plasma membrane, as shown by atomic force microscopy experiments which demonstrated that the budding of EVs at the plasma membrane of stem cells occurs at rates equal to their production.^25^ Moreover, findings of earlier electron microscope and electron microscopy experiments also prove this mode.^26^, ^27^, ^28^, ^29^ There may exist other modes of EXOs biogenesis. Recent researches illustrate that EXOs can be released in delayed by deep invaginations of certain cell types at the plasma membrane, which are indistinguishable from MVBs by conventional transmission electron microscopy.^30^, ^31^, ^32^ These intracellular plasma membrane–connected compartments (IPMCs) form a continuum with the extracellular milieu via necks, where vesicles can be stored and released in a pulsatile form.^30^

Exosomes biogenesis and release is regulated by several factors.

1. Rab proteins determine organelle membrane identity and mediate organelle dynamics^33^ but also regulate EXOs biogenesis that take place via endosomes and the plasma membrane. Rab27 proteins can mediate MVB maturation and traffic to the plasma membrane^34^, ^35^ and regulate plasma membrane PIP2 dynamics to organize microdomains involved in budding,^28^ together with Rab35.^36^ Rab11 proteins may affect EXOs release via a calcium‐induced homotypic MVBs fusion/maturation process.^37^
2. EXOs release can be promoted by the binding of the vacuolar protein sorting factor 4 (Vps4) and the endosomal sorting complex, required for transport (ESCRT), to ubiquitination protein. The ESCRT machinery contributes to membrane deformation, sealing and repairing in a wide array of processes that include MVB biogenesis.^38^ Therefore, EXOs biogenesis is likely to be ESCRT‐dependent,^39^ although this mechanism depends on other factors such as VPS4 ATPase.^38^, ^40^
3. EXOs biogenesis is also regulated by the Ral family of small GTPases. Their inhibition produced accumulation of MVBs near the plasma membrane and a marked reduction in secreted EXOs and EXOs marker proteins.^41^ The small GTPase Arf6 and the phospholipase PLD2 of the Ral family are also implicated in promoting EXO biogenesis.^42^ PLD2 seems to be dependent on a pair of exosomal scaffolds, syntenin and Alix.
4. Autophagy‐related (Atg) proteins coordinate initiation, nucleation, and elongation during autophagosome biogenesis. A decrease in EXOs secretion was observed in cancer cells lacking ATG5,^43^ whereas secretion of EXOs and EXOs related proteins increased in CRISPR/Cas9‐mediated neuronal cells knockout of Atg5.^44^
5. UV radiation, oxygen‐free radical stimulation, changes in calcium levels or cholesterol content may all contribute to variations in EXOs secretion.^45^, ^46^

### 2.2 Structure and composition

Exosomes, released by various types of cells to the extracellular space, are small (30‐150 nm) lipidic vesicles (LVs).^35^, ^47^, ^48^, ^49^, ^50^, ^51^ Since they are approximately 5‐ to 10‐fold smaller than the wavelength of visible light, they can be detected by conventional fluorescence microscopy when fluorescently labelled instead of conventional light microscopy.^28^ Cryo‐electron microscopy shows that EXOs display a spheroid morphology, although a small proportion has multiple membranes or presents elongated, tubule‐like morphologies. The latter may be generated by biological processes, by physical force‐induced fragmentation and mechanical resealing.^52^ EXOs have a density of ~1.1‐1.2 g/mL.^53^ Homogeneity is affected by protein, lipid ratio, expression of a specific single exosomal cargo protein^28^, ^29^ or exosomal metabolic pathways (eg, as a consequence of hydroxyapatite crystallization by osteogenic EXOs^54^).

Several tetraspanin proteins are highly enriched in EXOs, for example CD81, CD82, CD37 and CD63, with CD63 and CD81 are the least and the most enriched in B lymphocytes, respectively.^55^ In general studies, the successful isolation of EXOs from ADSCs is based on the detection of EXO markers (CD9, CD63 and CD81). Since that initial report, CD81 and CD63 have come to be among the most commonly used exosomal marker proteins, together with CD9,^56^ also detected in large vesicles.^57^ EXOs also have other transmembrane signal proteins acting as signal molecules,^58^ and carry cytokines, fibronectin, tenascin C and other extracellular matrix proteins (ECM). These suggest they participate in multiple signal platforms for autocrine and paracrine signaling.^59^, ^60^, ^61^ The inner cortex of exosomes is rich in scaffolding proteins Syntenin and Alix. In addition, a large number of tetrameric associated chaperones, including major histocompatibility complex (MHC),^53^, ^55^ immunoglobulin superfamily member 8 (IGSF8),^62^ intercellular adhesion molecule‐1 (ICAM‐1),^63^ syndecans 1 (SDC1) ^64^ and integrins,^65^ are also present in the endothelium of EXOs. The presence of heat shock proteins (HSPs) in EXOs was first reported by Mathew et al.^66^

Exosomes membrane riches (or riched) in cholesterol and sphingomyelin, with a small amount of lecithin and phosphatidylethanolamine.^67^ Also, the outermost surface of the EXOS consists of a glycan canopy, interrogated by lectin panning and other approaches.^68^, ^69^

Exosomes also contain nucleic acids include single‐stranded, genomic double‐stranded, mitochondrial or reverse‐transcribed complementary DNAs.^70^, ^71^, ^72^ RNAs include microRNA, ribosomal RNA and long non‐coding RNA, which can be transferred in functional form to other cells and tissues.^73^, ^74^, ^75^, ^76^

Adipose tissue is not only a reservoir of fat, but also an indispensable endocrine and immune organ. EXOs have been found in adipose tissue,^77^ adipocytes^78^, ^79^ and adipose‐derived mesenchymal stem cells (AMSCs).^80^ EXOs derived from ADSCs were larger than common EXOs,^81^ but expression of EXOs markers such as CD63 and HSP‐70 was positive, indicating that the size range of EXOs could be changed with different cell types. Adipocyte‐derived EVs distributed into large extracellular vesicles (lEV) and small extracellular vesicles (sEVs), respectively,^48^ with different protein composition. LEVs presented a high content of phosphatidylserine, whereas sEVs were rich in cholesterol, which also confirmed that lipid composition depends on cell source.^48^, ^82^

### 2.3 Isolation and identification technology

Common methods used to characterize EXOs include traditional fluorescence microscopy,^28^ super‐resolution microscopy,^83^ dynamic light scattering (DLS), nanoparticle tracking analysis (NTA), tunable resistive pulse sensing (TRPS) and single‐particle interferometric reflectance (SPIR). The latter can detect the presence and abundance of specific lipids, proteins, carbonic acids and carbohydrates.^84^

Exosomes are enriched by differential centrifugation, size‐exclusion chromatography, field flow fractionation, microfluidic filtration or contact‐free sorting immunoaffinity enrichment. Common methods for detecting EXOs‐labelled proteins include conventional protein analysis (Western blotting and ELISA, mass spectrometry), flow cytometry and newer protein analysis techniques, such as micro particle flow cytometry, micro‐nuclear magnetic resonance, nanoplasmonic EXO (nPLEX) sensor, integrated magnetic‐electrochemical EXO (iMEX) sensor and ExoScreen.^85^ Of these, differential centrifugation not only obtains more EXOs, but also avoid the influence of polyethylene glycol when using transmission electron microscopy.^86^

## 3 PHYSIOLOGICAL PROCESS AND MECHANISM OF WOUND HEALING

Wound healing is a complex dynamic physiological process, which can be generally divided into four stages: haemostasis, inflammation, proliferation and remodelling.^87^ Initial injury causes endothelial damage and basement membrane exposure, along with subsequent spillover of blood components. The immediate response to injury is vasoconstriction caused by the release of thromboxane and prostaglandins. Meanwhile, platelets adhere to exposed collagen and release the contents of their granules, whereas tissue factor activates both platelets and coagulation cascades.^88^ Blood clots formed by collagen, platelets, thrombin and fibronectin not only control haemorrhages but protect the wound and provide matrix and soluble factors to promote adhesion. They also concentrate growth factors that serve as wound healing scaffolds.^89^, ^90^ Blood clots also appear to be inducers of cell lineage differentiation during wound healing.^91^, ^92^, ^93^

During the inflammatory phase, vasodilation and capillary permeability results in oedema. Bone marrow‐derived immune cells prepare for wound healing by clearing pathogens, apoptotic cells, cell debris and damage mechanisms at the wound site.^87^ Cytokines and other factors attract granulocytes to wounds.^94^, ^95^ Subsequently, neutrophils digest debris and injured tissues by secreting proteases. And clear microbial pathogens through oxygen‐dependent mechanisms. Local monocytes also migrate into the wound and become macrophages, which can phagocytose apoptotic cells and cell debris and secrete a large number of growth factors.^96^ Lymphocytes are also involved in inflammation. Interestingly, CD4^+^ T cells are associated with healing, whereas CD8^‐^T cells negatively affect this process.^97^ Inflammation eventually promotes transformation of M1 macrophages to M2 macrophages.^98^

In the proliferative phase, re‐epithelization occurs. This relies on migration of epithelial cells from the wound margins and any remaining adnexal structures in the dermis. Epithelial migration and proliferation continue until the wound is completely covered and an intact epithelial barrier is reestablished.^99^ M2 macrophages promote tissue regeneration and a mass production of extracellular matrix by regulating the proliferation and migration of keratinocytes, fibroblasts and endothelial cells.^91^ Fibroblasts begin to secrete high levels of immature collagen type III into the matrix.^100^

In the last remodelling period, fibroblasts continue to secret collagen^101^, ^102^ and over time collagen type III decreases and is replaced by collagen type I. Collagen fibres gradually become organized and the tensile strength of the wound increases.^103^ Collagen breakdown and structural adjustment of the neonatal extracellular matrix results in reduced wound thickness, degradation of newly formed capillaries and narrowing of the wound edge through contraction of the subvascular connective tissue.^87^, ^93^, ^104^

## 4 ADSCs‐EXOs MEDIATE WOUND HEALING

### 4.1 Regulations of immune response and inflammation

Inflammation is the body's self‐defence mechanism in response to harmful stimuli. Wound healing can be delayed by chronic and excessive inflammation, therefore a well‐regulated inflammation guarantees wound healing.^105^ Activated T regulatory cells can promote wound healing by reducing both production of interferon alpha (IFN‐α) and aggregation capacity of M1 macrophages.^106^ ADSCs‐EXOs play an immunosuppressive role by reducing IFN‐α secretion, thus inhibiting activation of T cells.^107^ Furthermore, ADSCs‐EXOs contain immunoregulatory proteins such as TNF‐a, macrophage colony‐stimulating factor (MCSF) and RBP‐4.^108^ The role of ADSCs‐EXOs in promoting monocyte differentiation into M1 macrophages was confirmed by Kranendonk et al^108^ It was also found that miR‐155 in ADSCs‐EXOs can induce adipocyte‐derived macrophages from obese mice to differentiate into M1, causing chronic inflammation with an imbalance in the M1‐to‐M2 macrophage ratio in adipose tissue.^109^ ADSCs‐EXOs can also up‐regulate the expression of macrophage inflammatory protein‐1α and monocyte chemoattractant protein‐1, promoting early inflammation.^110^

### 4.2 Promoting angiogenesis in wounds

Angiogenesis provides blood supply for wound healing, facilitating the transport of nutrients and metabolic waste products.^89^ ADSCs‐EXOs promote the proliferation and migration of vascular endothelial cells, thereby enhancing angiogenesis.^111^ Human adipose stem cells (hADSCs)–derived EXOs are rich in miRNA‐125a and miRNA‐31, which can be transferred to vascular endothelial cells to stimulate proliferation and promote angiogenesis. Transfer of miR‐125a to endothelial cells has been demonstrated in vitro and in animal experiments.^112^ MSCs‐EXOs could inhibit expression of angiogenesis inhibitor (DLL4), thus promoting migration and sprouting vascular endothelial tip cells. Transfer of miRNA‐31 to endothelial cells has also been shown,^113^ where hADSCs‐EXOs inhibited expression of the anti‐angiogenesis gene HIF1 in vascular endothelial cells, promoting migration and enhancing angiogenesis in human umbilical vein endothelial cells. ADSCs‐EXOs may also promote the survival of skin flaps and increase capillary density, playing a role in repairing ischaemia‐reperfusion injury.^114^

### 4.3 Speeding up proliferation and re‐epithelialization of skin cells

During the proliferative phase, fibroblasts proliferate to produce ECM, whereas epithelial cells proliferate and migrate towards the wound centre to promote wound healing. Thus, proliferation and re‐epithelization of skin cells are important for skin regeneration.^89^ ADSCs‐EXOs are internalized by fibroblasts and stimulate proliferation, migration and collagen synthesis in a dose‐dependent manner.^115^ ADSCs‐EXOs accelerate cutaneous wound healing by optimizing fibroblast properties, as shown in in vivo experiments.^113^ Finally, hADSCs‐EXOs up‐regulated 199 miRNAs and down‐regulated 93 miRNAs to promote dermal fibroblast proliferation and differentiation that sped up skin regeneration.^116^

### 4.4 Regulating collagen remodelling to inhibit scar hyperplasia

**FIGURE 2:**
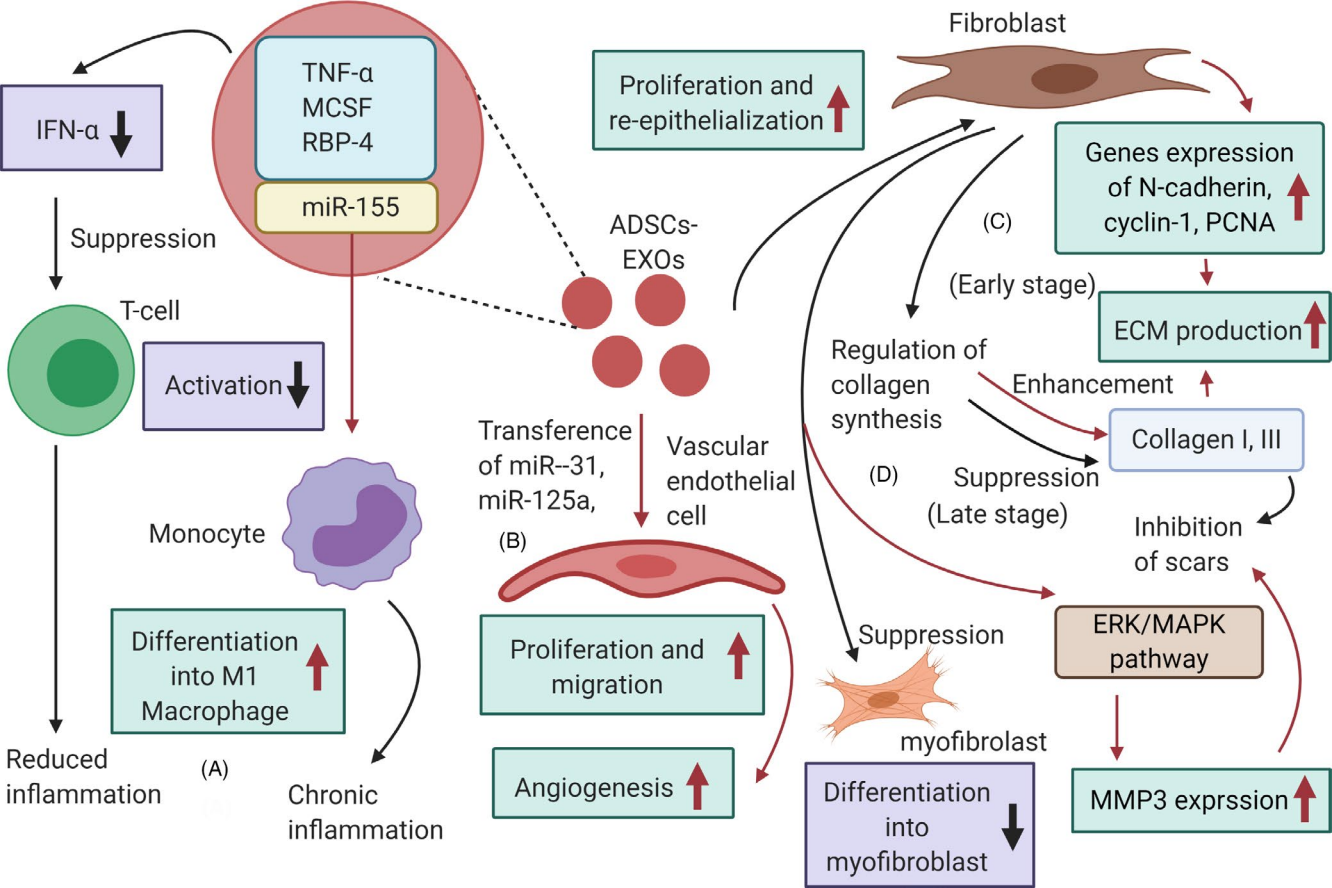
Mechanisms by which ADSCs‐EXOs may promote wound healing. (A) adipose‐derived stem cells (ADSCs)‐EXOs contain immunoregulatory proteins and reduce the secretion of IFN‐α, subsequently inhibiting activation of T cells, resulting in reduced inflammation. Additionally, miR‐155 in ADSCs‐EXOs can induce monocyte differentiation into M1 macrophages, causing chronic inflammation; (B) ADSCs‐EXOs can transfer miRNA‐125a and miRNA‐31 to vascular endothelial cells, stimulating proliferation and migration to promote angiogenesis; (C) In the early stages, ADSCs‐EXOs may promote proliferation, migration and collagen synthesis in fibroblasts, stimulating N‐cadherin, cyclin‐1, PCNA and collagen I, III expression and increasing ECM production; (D) in late stages, ADSCs‐EXOs prevent the differentiation of fibroblasts into myofibroblasts, and reduce scarring by inhibition of the formation of collagen and activation the ERK/MAPK pathway to increase MMP3 expression. Abbreviations: ECM, extracellular matrix; MCSF, macrophage colony‐stimulating factor

Scar hyperplasia is a morphological and histopathological change of skin and soft tissue after wound healing. Severe trauma and extensive burn usually lead to scar proliferation, affecting aesthetic appearance and impairing organ function.^89^ ADSCs‐EXOs can regulate collagen remodelling to inhibit scar hyperplasia. In an early stage, EXOs promote collagen remodelling through synthesis of type Ⅰ and Ⅲ, whereas they reduce scarring in the late stage by inhibiting collagen formation.^115^ In addition, ADSCs‐EXOs can stimulate the reconstruction of extracellular matrix by regulating fibroblast differentiation and gene expression, thereby promoting wound healing and preventing scar proliferation. Wang et al^117^ found that ADSCs‐EXOs prevented the differentiation of fibroblasts into myofibroblasts but increased the ratio of transforming growth factor‐β3 (TGF‐β3) to TGF‐β1 in vivo. ADSCs‐EXOs also increased the matrix metalloproteinases‐3 (MMP3) expression in skin dermal fibroblasts, resulting in a high ratio of MMP3 to tissue inhibitor of matrix metalloproteinases‐1 (TIMP1). This is beneficial for the remodelling of extracellular matrix (ECM), reducing scaring. Instead, in diabetic mice, ADSCs‐EXOs promoted collagen deposition, which increased in the late stage of wound healing.^118^ However, this leads to scar hyperplasia, which is not conducive to healing.^18^ These controversial results may be due to the complex function of collagen and EXOs during different stages of wound healing. More studies on the effect of ADSCs‐EXOs on collagen deposition and their association with scar proliferation need to be performed (Figure 2).

## 5 CLINICAL APPLICATION OF ADSCs‐EXOs FOR WOUND REPAIR AND TREATMENT

### 5.1 Improving fat grafting

Fat grafting is used in cosmetic surgery, for example in the treatment of hemifacial atrophy, depressed scars and breast reconstruction.^119^, ^120^ Adequate blood supply in the early stage after transplantation is required for successful grafting^121^ which highlights the importance of promoting angiogenesis. ADSCs‐EXOs can promote proliferation and migration of vascular endothelial cells, thus promoting angiogenesis.^122^ They can also raise fat graft volume retention in adipose‐derived mesenchymal stem cells. Indeed, EVs from ADSCs could improve fat graft volume retention by stimulating angiogenesis and regulating inflammatory response^123^ and EXOs were found comparable to source ADSCs in fat graft retention, up‐regulating early inflammation and angiogenesis ^110^; thus, it is clear that ADSCs‐EXOs may play an important role in the improvement of fat grafting in the clinic. Lastly, EXOs from hypoxia‐treated human adipose‐derived mesenchymal stem cells possessed a higher capacity to enhance angiogenesis in fat grafting.^124^ The latter may help develop new strategies to improve the survival of fat grafts.

### 5.2 Promoting wound healing of diabetic patients

According to the World Health Organization (WHO), the number of diabetes patients will reach 592 million by 2035,^125^ and one of the most challenging complications of diabetes mellitus (DM) is delayed wound healing.^126^ Lack of ideal treatments among all available ones appeals many scientists to develop new therapies.^127^ Wang et al^118^ demonstrated that ADSCs‐EXOs could promote wound healing in diabetic mice by promoting angiogenesis, proliferation and migration of fibroblasts and collagen synthesis. The capacity of ADSCs‐EXOs to promote wound healing in diabetic foot patients was also reported.^128^ The latter study showed that ADSCs‐EXOs can simultaneously express antioxidant receptors (Nrf2), laying an experimental foundation for clinical application of EXOs to treat chronic diabetic wounds. In diabetic mice, ADSCs‐EXOs also promoted increased collagen deposition in the late stage of wound healing,^118^ but excessive collagen deposition may be unfavourable as it leads to scar hyperplasia.^18^ This controversy remains unsolved and therefore more research is required on the effect of ADSCs‐EXOs on collagen deposition and their association with scar proliferation.

### 5.3 EXOs as a carrier and combined scaffold for treatment

The natural biocompatibility and cell‐targeting features equip EXOs for carring (delivering) drugs.^18^ To stabilize their concentration following local application, hydrogel or fibrin can be used as scaffolds to delay EXOs release and enhancing their wound healing ability.^18^ Shilan et al^129^ used EXOs loaded in alginate gel as a bioactive scaffold in an in vivo study. This showed that this active wound dressing technique could significantly promote wound healing, collagen synthesis and local angiogenesis, providing a new strategy for the composite structure of alginate hydrogel to speed up the healing process.

### 5.4 Promoting scarless cutaneous repair

Scar formation after skin wounds have healed is an intractable medical problem, affecting both aesthetic appearance and organ function.^89^ In murine incisional wounds, intravenous injection of ADSCs‐Exos decreased the size of scars, increased the ratio of collagen III to collagen I and regulated fibroblast differentiation and gene expression^117^; thus, ADSCs‐EXOs may be a new treatment for scarless cutaneous repair.

## 6 DISCUSSION

**FIGURE 3:**
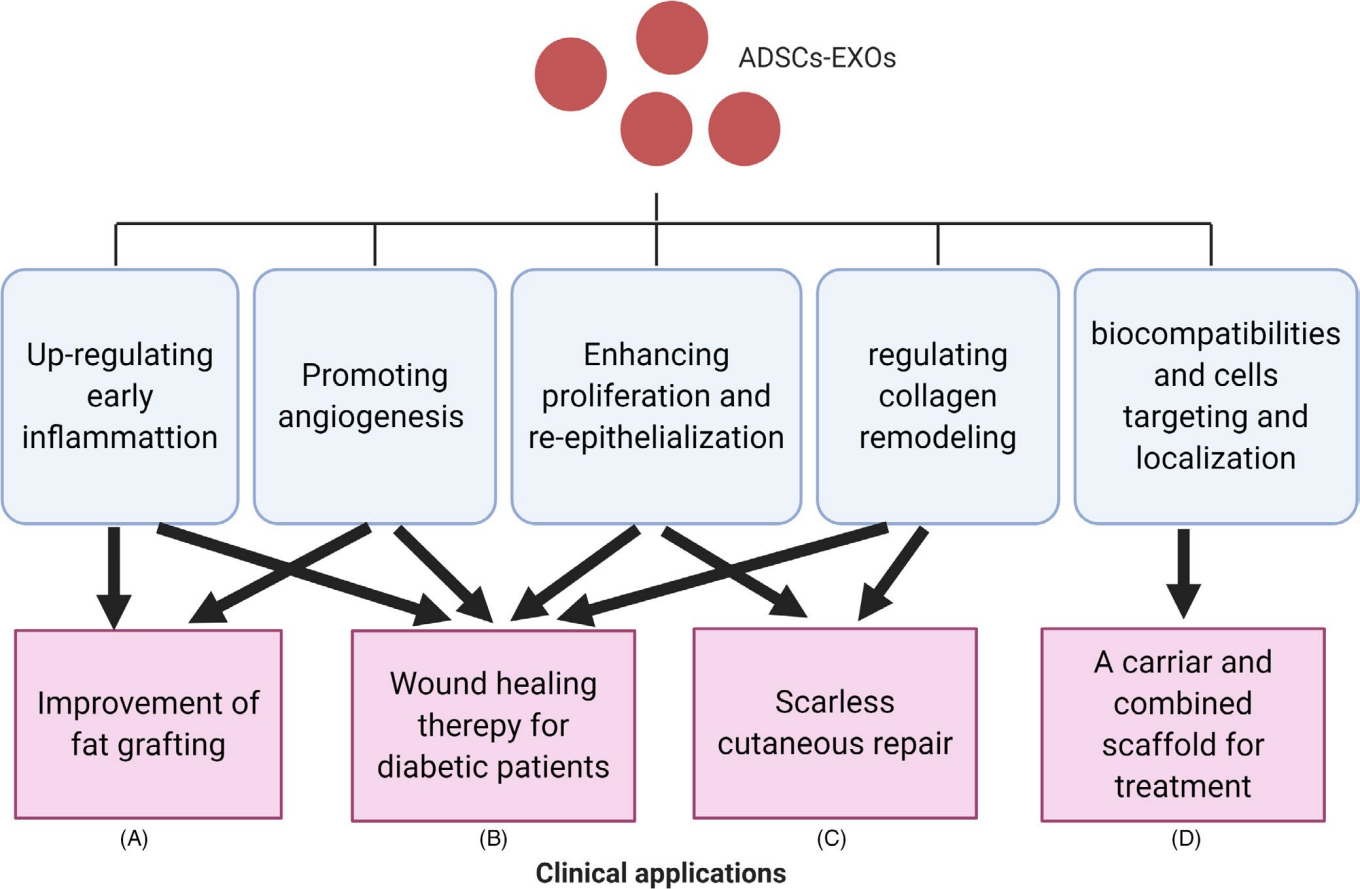
Potential clinical applications of adipose‐derived stem cells (ADSCs)‐EXOs: improvement of fat grafting (A), wound healing therapy for diabetic patients (B), scarless repair (C) and carrier and combined scaffold for treatment (D)

Adipose‐derived stem cells‐EXOs have a great potential in the clinic for wound repair and regeneration (Figure 3). They can participate in the regulation of the immune response and wound inflammation and promote angiogenesis by transferring miRNA‐125a and miRNA‐31 to vascular endothelial cells. Also, ADSCs‐EXOs can stimulate the proliferation of fibroblasts and keratinocytes and regulate collagen remodelling. This inhibits scar hyperplasia by activating the ERK/MAPK pathway that regulates the secretion of related cytokines. These properties make them an optimal tool to improve fat grafting application, promote wound healing of diabetic patients and scarless cutaneous repair and also to act as a carrier for combined scaffolds used for treatment. Recently, more attention is given to self‐derived and free‐cell auxiliary agents, especially ADSC‐Exos. Oral wound repair may use free‐cell therapies to promote oral mucosa defects healing^130^ and reduce inflammatory process in wound after tooth extraction.^131^ Moreover, these therapies are also used in the healings of acute and chronic ulcers,^132^ postoperative hand wounds,^133^ chronic lower‐extremity wounds.^134^ We have every reason to believe there is more potential in the use of ADSC‐Exos in free‐cell therapies to be discovered.

However, although their efficacy has been proved, the mechanism is not yet clear. There remains a burning question in this field about the pro‐ or anti‐cancer status of ADSCS‐EXOs. Thus, safety and efficacy of ADSCs‐EXOs cannot be guaranteed. The problems of lacking of easy process of extraction and purification of EXOs and standard methods for identifying EXOs from specific cell sources also need to be solved. To better isolate and identify ADSCS‐EXOs and understand their mechanism of action, further research is needed in the expect of more efficient ADSCs‐EXOs products and boarder applications in clinical practice.

## 7 CONCLUSION

In summary, ADSCs‐EXOs are a highly promising therapeutic for wound repair and regeneration. In the wound, ADSCs‐EXOs modulate immune responses and inflammation, promote angiogenesis, accelerate proliferation and re‐epithelization of skin cells and regulate collagen remodelling which inhibits scar hyperplasia. ADSCs‐EXOs can improve fat grafting, promote wound healing of diabetic patients and act as a carrier and combined scaffold for treatment, leading to scarless cutaneous repair. ADSCs‐EXOs have a board applications in clinical practice and are likely to achieve the best fictionally skin wound healing.

## CONFLICT OF INTEREST

The authors declare no conflict of interest.

## Data Availability

Data sharing is not applicable to this article as no new data were created or analysed in this study.
